# XPERT^®^ breast cancer STRAT4 as an alternative method of identifying breast cancer phenotype in Cape Verde (preliminary results)

**DOI:** 10.3332/ecancer.2023.1530

**Published:** 2023-04-11

**Authors:** Pamela C C Borges, Hirondina B Spencer, Carla Barbosa, Victor Costa, Antónia Furtado, Maria Conceição Leal, Carlos Lopes, Dylan Ferreira, André Lopes Carvalho, Isabel Dos-Santos-Silva, Lúcio Lara Santos

**Affiliations:** 1 Laboratório Biologia Molecular, Hospital Universitário Agostinho Neto, Praia, Plateau 112, Cabo Verde; 2 Hospital Universitário Agostinho Neto, Praia, Plateau 112, Cabo Verde; 3 IMP Diagnostics, Molecular and Anatomic Pathology Lab, 4150-146, Porto, Portugal; 4 Anatomia Patológica, Instituto Português de Oncologia, 4200-072, Porto, Portugal; 5 Unilabs | Laboratório Anatomia Patológica, 4250-170, Porto, Portugal; 6 Experimental Pathology and Therapeutics Group, Research Center of IPO Porto (CI-IPOP)/RISE@CI-IPOP (Health Research Network), Portuguese Oncology Institute of Porto (IPO Porto)/Porto Comprehensive Cancer Center (Porto.CCC), 4200-072, Porto, Portugal; 7 International Agency for Research in Cancer, 69007, Lyon, France; 8 London School of Hygiene & Tropical Medicine, WC1E 7HT, London, UK; 9 Surgical Oncology Department, Portuguese Institute of Oncology, 4200-072, Porto, Portugal

**Keywords:** breast cancer, alternative diagnosis, GeneXpert, Xpert^®^ STRAT4

## Abstract

**Introduction:**

Breast cancer (BC) is a public health problem in developing countries, including Cape Verde. Immunohistochemistry (IHC) is the gold standard technique used for BC phenotypic characterisation to support efficient therapeutic decisions. However, IHC is a demanding technique that requires knowledge, trained technicians, expensive antibodies and reagents, controls, and results validation. The low number of cases in Cape Verde increases the risk of expiring the validity of the antibodies, and manual procedures often jeopardise the quality of the results. Thus, IHC is limited in Cape Verde, and an alternative technically easy solution is needed. A point-of-care messenger RNA (mRNA) STRAT4 BC assay to assess estrogen (ER), progesterone (PR), hormone growth factor 2 receptor (HER2), and Ki67, using the GeneXpert platform, has been recently validated on tissues from internationally accredited laboratories, showing excellent concordance with IHC results.

To assess whether this technology can be implemented in Cape Verde to guide BC treatment we decided to study the level of agreement between the findings yielded by BC STRAT4 and the results are the same cases obtained by IHC.

**Methods:**

Formalin-fixed and paraffin-embedded (FFPE) tissue samples from 29 Cabo Verdean BC patients diagnosed in Agostinho Neto University Hospital were analysed by applying IHC and BC STRAT4 assay. The time between sample collection and pre-analytic procedures is unknown. All the samples were pre-processed in Cabo Verde (fixed in formalin and embedded in paraffin). IHC studies were performed in referenced laboratories in Portugal. STRAT4 and IHC result concordance was assessed by calculating the percentage of results agreement and Cohen’s Kappa (K) statistics.

**Results:**

STRAT4 assay failed in 2 out of the 29 analysed samples. Of the 27 successfully analysed samples, STRAT4/IHC results for ER, PR, HER2, and Ki67 were concordant in 25, 24, 25, and 18 cases, respectively. Ki67 was indeterminate in three cases, and PR was indeterminate once.

The percentage of agreement between STRAT4 and IHC results for ER, PR, HER2, and Ki67 was 92.59%, 92.31%, 92.59% and 81.82%, respectively. The Cohen’s K statistic coefficients for each biomarker were 0.809, 0.845, 0.757 and 0.506, respectively.

**Conclusions:**

According to our preliminary results, a point-of-care mRNA STRAT4 BC assay may be an alternative in laboratories unable to provide quality and/or cost-efficient IHC services. However, more data and improvement on sample pre-analytic processes are required to implement this BC STRAT4 Assay in Cape Verde.

## Background

Breast cancer (BC) is nowadays a public health problem in several developing countries, with increasing incidence and mortality rates [1–4].

Despite limited epidemiologic data, the rising incidence of BC in Africa is being driven by increasing life expectancy, improved control of infectious diseases, westernisation of lifestyles associated with changes in reproductive behaviour (i.e., delayed age at first birth, fewer children and reduced breastfeeding duration), changes in diet, increase of alcohol intake, decrease in physical activity, and increase in body weight. Other determinants unique to women of African origin have been suggested, such as the use of skin lighteners and increased exposure to hormone modulators in skincare and hair products, often used by women of African descent [3–8].

The public health response has been insufficient to control the disease burden, with many low- and middle-income countries having inadequate capacity to provide high-quality and accessible cancer programs. This deficient response threatens public health, economic growth, and the achievement of the UN Sustainable Development Goals [9]. Sub-Saharan Africa needs early diagnosis and BC molecular characterisation to promote more efficient therapeutic decisions and to provide adequate curative treatment and palliative care services [4, 10, 11].

Cape Verde’s population is half a million. According to the 2021 census, 250,262 are men and 247,801 women [12]. The population is young; however, the fertility rate has been decreasing over the years while life expectancy has been increasing. In 2019 the average life expectancy for women reached 80.5 years old [12]. In Cape Verde non-communicable diseases are the main cause of death and, among them, cancer is the second cause of death in the country [13]. BC is nowadays the second most common cancer among females. The incidence rate is not well known however, WHO estimates that BC incidence is 18/100,000 habitats [14].

BC is molecularly classified into four main subtypes: luminal A, luminal B, luminal B hormone growth factor 2 receptor (HER2)-like, HER2 enriched, and triple negative. Each BC cancer subtype has specific molecular traits and requires specific treatments [15–21]. In this context, one of the most important aspects to guarantee rapid and efficient BC therapeutic decisions and treatment is the study of the tumor’s phenotype. Immunohistochemistry (IHC) is the standard and most applied technique, and it is used to assess biomarkers such as estrogen (ER) and progesterone (PR) hormone receptors, HER2, and Ki67 proliferative marker. Frequently, further studies are required to assess the presence of HER2 and that is done by applying hybridisation techniques such as FISH (fluorescence in situ hybridization) or SISH (Silver in situ hybridization) [22].

Despite being widely used, IHC is a technique that requires considerable laboratory resources and technical expertise to ensure that it is carried out appropriately and its findings correctly interpreted, including properly trained technicians, expensive antibodies and reagents, use of proper controls and results validation.

In Cape Verde, IHC was implemented. However, its optimisation is still an ongoing process. The IHC is performed manually, given the small number of cases per year, with difficulties in acquiring antibodies, and associated reagents, and the expiry date is frequently exceeded. Thus, alternative techniques, easier to perform, easier logistically and possibly cheaper to support BC diagnosis, are required. Thus, the training of technicians will be easier, and the costs will eventually be lower. For these reasons, this methodology may empower the health services on the islands that have pathology laboratories.

Several studies showed an association between *ESR1, PGR, ERBB2, MKI67* messenger RNA (mRNA) quantitative measurements and ER, PR, HER2 and Ki67, respectively [23–25]. The new Xpert BC STRAT4 is a potentially affordable, automated, quick and easy-to-use assay based on quantitative, real-time, polymerase chain reaction (RT-pPCR) [25–28]. In Cape Verde this platform is already widely available and used across several islands for diagnosis of infectious diseases and the handling of expiry dates is more manageable since the technicians are not dealing with several reagents to produce a result.

To be implemented in Cape Verde, validation with local pre-analytical conditions is required. Thus, we decided, to study the level of agreement, in a series of BC cases, between the STRAT4 and IHC in the same BC cases.

## Methods

### Study design and samples

This is a retrospective study designed to investigate result concordance between the Xpert**^®^** BC STRAT4 assay and the standand IHC/FISH or SISH method. To perform this study samples from 29 patients consecutively registered with BC at Agostinho Neto University Hospital (ANUH) from 2017 to 2021 in which their molecular phenotype had been studied by IHC. All the samples were formalin-fixed and paraffin-embedded (FFPE) tissues derived from surgically excised breast tumors and underwent pre-analytical procedures at ANUH Pathology Lab. The blocks are stored at room temperature, in paper boxes. The storage time of these blocks ranged from a few months to 6 years. Each sample was tested using both Xpert BC STRAT4 assay and IHC/FISH or SISH was again performed to determine BC subtype.

### XPERT® BC STRAT4 assay and software

Tests using GeneXpert**^®^** (GX) BC STRAT4 assay were performed at the Microbiology Lab and Experimental Pathology and Therapeutics Lab of IPO-Porto. For each specimen, a 4 μm thick sectioned slide was stained with hematoxylin and eosin and used to mark the tumor area and estimate the size and the percentage of tumor content.

Unstained slides containing tumor samples of 10 μm thickness were micro-dissected into a 1.5 mL tube. Posteriorly FFPE samples were lysed using FFPE-Lysis Kit (GX FFPE-LYSIS-CE-10) following the concentrated extraction protocol: 260 uL of FFPE lysis buffer, 5 uL proteinase K, 5 seconds maximum speed vortex. The lysate was incubated at 80°C for 30 minutes; After incubation, samples were vortexed for 5 seconds at top speed; 260 uL of ethanol (>95%) was added and samples were vortexed for 15 seconds. For each sample, 520 uL of lysate was loaded into the sample chamber of the Xpert**^®^** BC STRAT4 cartridge which was then placed into the GX machine to perform the assay (RNA extraction, purification, and RT-qPCR analysis). BC STRAT4 assay assesses *ESR1, PGR, ERBB2*, and *MIKi67* mRNA expression levels.

GX DX Software was used to analyse results from GeneXpert BC STRAT4 assay. Cytoplasmic FMR 1-interacting protein 1 (CYFIP1) is used as an internal reference gene and all target genes (*ESR1, PGR, ERBB2, MKI67*) mRNA measurements are normalised against *CYFIP1* mRNA measurements.

Cycle threshold (Ct) values for the reference gene (*CYFIP1*) and target genes (*ESR1, PGR, MKi67*, and* ERBB2*) are measured simultaneously. Delta Ct (dCt = reference gene Ct minus target gene Ct) for each marker is calculated using the *CYFIP1* Ct as reference. Delta Ct assays cutoffs are ‘−1’ for *ESR1* and *ERBB2*, ‘−3.5’ for *PGR* and ‘−4’ for *MKi67*.

To prevent false negatives, a minimum *CYFIP*1 Ct was implemented as 31. dCt values for each marker is then compared to pre-specified Ct and dCt cutoffs to classify *ESR1, PGR, MKi67*, and *ERBB2* mRNA expression as POSITIVE, NEGATIVE, INDETERMINATE, INVALID, or ERROR. Markers are INDETERMINATE (applicable for PGR and MKi67) when dCt value is below the specified cutoff value and *CYFIP1* Ct is greater than 31 and INVALID when *CYFIP1* Ct is greater than 35.

### Immunohistochemistry

The IHC/FISH or SISH study was carried out in two European laboratories where, previously, there was a contract by the ANUH to carry out these procedures. For this reason, we decided to repeat the study of seven cases randomly chosen, to verify if there were differences in their classification. If there were no differences, the cases would be studied together regardless of the laboratory where the IHC studies had been carried out, as was the case. Before IHC, the histological revision was made by expert pathologists (CB (Carla Barbosa), AF (Antónia Furtado), MCL (Maria Conceição Leal) and CL (Carlos Lopes)), following WHO 2012 classification. The IHC was performed using monoclonal ER for ER receptors (clone 6F11, 1:150, Novocastra Laboratories, Leica Microsystems), PR for PR receptors (clone 16, 1:200 Novocastra Laboratories, Leica Biosystems), Ki-67/MIB-1 (1:200, DAKO (Glostrup, Denmark)) and HER-2 (1:100, HercepTest DAKO). Bound antibodies were detected using 3,3-diaminobenzidine chromogen from Refine Polymer Detection Kit (Leica Microsystems). To counterstain the slides, we used Harris’ hematoxylin.

Results evaluations were made having positive and negative tissue controls and following ASCO/CAP (The American Society of Clinical Oncology/College of American Pathologists) guidelines and recommendations for hormonal receptors. Ki-67 cut-off was defined at 15%. Proliferative Ki-67 index was considered high when nuclear expression in tumor cells was ≥15%. ER and PR were considered positive if more than 1% of nuclei were positive for hormone receptors. Interpretations for HER2 IHC were made according to the 2018 ASCO-CAP HER2 Test Guideline Recommendation [29]. Cases interpreted as 0 or 1+ were considered negative, 2+ were equivocal (in these cases, the study was carried out by FISH or SISH to classify them.) and 3+ cases were considered positive.

According to IHC results and, following ESMO, BCs cases were molecularly classified as luminal A- like (ER+ and/or PR+, HER2−, K-67 < 15%), luminal B- like (further classified according to HER2: HER2 negative: ER+ and/or PR+ and HER2−, K-67 ≥ 15%, or; or ER+ and/or PR− and HER2−, K-67 ≥ 15%, HER2 positive: ER+ and/or PR+, HER2+); HER2 positive (ER− and/or PR− and HER+) and triple-negative (negative RE, RP, and HER2) [30].

### GX molecular BC classification

According to Janeva *et al* [31] methodology, we define a classification to determine the surrogate BC subtype. Thus, according to STRAT4 results, BC is luminal A when *ESR1* and/or *PGR* are positive, *ERBB2* is negative and *MKi67* is negative or indeterminate. BC is luminal B if *ESR1* and/or *PGR* are positive, *ERBB2* negative or *MKi67* positive. It is classified as luminal B HER2+ when *ESR1* and/or *PGR* are positive, *ERBB2* is positive or *MKi67* is positive. BCs are HER2 Enriched if *ESR1* and/or *PGR* are negative and* ERBB2* is positive. It is triple-negative when *ESR1* and/or *PGR* are negative; *ERBB2* is negative and *MKi67* may be positive or negative.

### Statistical analysis

The data was introduced and treated using Excel Software, version 16.43. Result concordance between STRAT4 and IHC/FISH methods was accessed by estimating the percentage of concordance as well as the Cohen’s Kappa (K) statistics. The Kappa (K) statistic numeric values are categorised into slight agreement (≤0.2), fair agreement (between 0.21 and 0.40), moderate agreement (between 0.21 and 0.40), substantial agreement (between 0.61 and 0.80) and almost perfect agreement (between 0.81 and 1.00). All measurements were associated with 95% confidence intervals (95% CI), compared using Fisher’s exact test and considered significant for *p* < 0.05.

### Ethics

This study was conducted according to international standards for good clinical practices. *Comitê Nacional de Proteção de dados de Cabo Verde* (Cabo Verde National Committee for Data Protection number 26/2021) and* Comitê de Ética de Cabo Verde* (Cabo Verde Ethics Committee number 03/2021) gave permission to realise studies regarding the molecular characterisation of BC tumors at ANUH. All participants provided written informed consent for researchers to access and test their tumor specimens.

## Results

The IHC/FISH or SISH and STRAT 4 results for each case can be found in a table included in the supplementary data (Table S1). STRAT4 assay failed in 2 out of the 29 analysed samples. The two STRAT4 assays that did not work displayed their results as invalid. According to the test report, the run passed all the probe check tests, and no errors, shifts, trends, or operator variabilities were detected. This indicates that the cartridge used was in perfect condition and there were no problems with the system. The test reports of these samples also revealed that *ESR1, PGR, ERBB2,* and *MKi67* results were invalid, and CYFIP1 internal control failed which suggests problems with the samples themselves. This was confirmed by the IHC report from the pathologist who highlighted significant pre-analytical issues on those samples; a severe fixation problem was detected.

From the 27 cases successfully analysed samples, Ki67 was indeterminate in three cases and PR was indeterminate in one. STRAT4/IHC results for ER, PR, HER2, and Ki67 were concordant in 25, 24, 25, and 18 cases, respectively (Table 1). It was not possible to access IHC and STRAT4 result concordance for Ki67 in five cases: Two Ki67 STRAT4 results were indeterminate, and three Ki67 IHC results were not reported. STRAT4 MKi67 was the biomarker with the most discordant results. It was discordant with IHC results in six samples and was indeterminate in three cases. STRAT4 PGR was indeterminate in only one sample and the pathologist reported that the FFPE tissue sample used in these tests had a low percentage of tumor material.

The percentage of agreement for ER, PR, HER2, and Ki67 was 92.59%, 92.31%, 92.59%, and 81.82%, respectively. The Cohen's K statistic coefficients for each biomarker were 0.809, 0.845, 0.757, and 0.506, respectively (Table 1).

A comparison between BC classification made from IHC and STRAT4 results was also made. There was 77.78% (21/27) concordance between the BC classification obtained from IHC and STRAT4 results (Table 2).

In six (22.6%) cases, all luminal B by IHC, were in disagreement with the classification obtained from STRAT4. In two cases, despite also being luminal B, HER2 expression was only detected in STRAT4 assay. Two other cases were shown to be triple negative in STRAT4. The last two cases were classified as luminal A in the STRAT4 assay. In two cases the results were invalid due to technical aspects related to the quality of the paraffin block. The table with the BC molecular classification in the series studied, based on IHC and STRAT4 assay results, is found in the supplementary data (Table S2).

## Discussion

BC molecular characterisation and sub-typing are extremely important to define and promote efficient therapeutic decisions and treatment. Being able to establish the molecular subtype of a patient’s BC accurately, quickly, and efficiently is crucial in guiding treatment decisions and, ultimately, increasing survival from the disease.

The standard technique used to characterise BC molecularly is IHC, despite being a delicate and prone to error technique that requires trained technicians and expensive reagents and antibodies. In Cape Verde, IHC is still in the implementation stage, and therefore the quality and efficiency of this technique’s performance still require monitoring. On the other hand, the acquisition of reagents is dependent on the financial constraints and importation difficulties that are common in island countries.

The GX (Cepheid) point-of-care technology is available and widespread in Cape Verde to diagnose infectious diseases, and its availability was reinforced with the SARS-CoV-2 (Severe acute respiratory syndrome coronavirus 2) pandemic.

BC STRAT4, a recent GX assay, is an automated, quick, and easy-to-use alternative technology that surrogates BC sub-typing. However, it is important to analyse STRAT4 efficiency, taking into consideration the way samples are processed during pre-analytical procedures, in the country.

This study was designed to evaluate the STRAT4 efficiency using FFPE BC tissue samples collected, processed, and stored in Cape Verde by comparing its results with results from IHC performed abroad. Comparison between our results from STRAT4 and IHC revealed high concordance for ER and PR results and substantial concordance for HER2 in agreement with similar studies [25–28]. Considering our results, as well as other similar results found in other published papers, the Xpert^®^ BC STRAT4 assay could be a potential solution to overcome IHC/FISH limitations and may help facilitate access to invasive BC testing in low-resource countries.

However, the concordance turned out to be moderate for Ki67, similar to the data that has been published [25–28, 32]. At the 13th Saint Gallen International BC Consensus, a surrogate classification of BC molecular subtypes by IHC was established [33]. The most controversial point was the difference between the luminal A and luminal B subtypes according to the Ki67 values [34]. Commonly, 14% is the Ki67 cut-off that has been established for differentiating BC subtypes; however, in later studies this value has been questioned and a cut-off of 20% has been proposed [30]. Recently, Escala-Cornejo *et al* [35] to identify the best Ki67 cut-off for determining luminal BC Subtypes using immunohistochemical analysis and PAM50 Genomic Classification propose that the Ki67 cut-off should be globally modified to >20%. For this biomarker, significant variability in concordance rate has been noted, although this is not unforeseen given the challenges associated with Ki67 IHC evaluation. However, the highest concordance rate (with STRAT4) was observed when the percentage of cells labeled with KI67 by IHC is greater than 20% [36]. It should be noted that classification into luminal A and luminal B does not necessarily imply a mandatory therapeutic change, except when HER2+, advanced TNM classification (Tumour, Lymph node, Distant metastasis) staging, or even a high histological grade.

According to the classification used by us to assess the surrogate BC subtype with the STRAT4, we found that the concordance with the classification performed by the IHC was 77.78%. The cases (22.6%) in which the molecular subtypes according to the IHC assay were divergent from the results obtained by the STRAT 4 assay, would imply different therapeutic protocols like what was observed in other series [31].

Our data showed that the proficiency of this assay, in our hands, can be improved, since we observed that BC tissue samples that had STRAT4/IHC discordant results had fixation problems. This reveals the need to introduce improvements to the local FFPE tissue sample pre-analytical handling procedures (optimisation of tissue sample fixation) to maximise the efficiency of IHC and the STRAT4 assay [28, 37].

Samples with scarce tumor material also had STRAT4 results that were discordant with IHC results. Therefore, FFPE tissues with scarce tumor material should be avoided, or it is crucial to make sure that enough tumor material is used. However, studies are validating the use of STRAT4 assay in fine needle aspiration biopsies [31, 38, 39].

A recent study compared the financial and time cost of BC biomarker analysis by IHC with that by the STRAT4 assay and concluded that BC biomarker analysis with STRAT4 has the potential to reduce the required human and capital resources in sub-Saharan African laboratories, leading to improved treatment selection and better clinical outcomes [40].

We consider it essential, before generalising its use in Cape Verde, to test the STRAT4 on a larger series with better pre-analytical quality and include the analysis of cost.

Considering our results, as well as results in other published papers, the Xpert^®^ BC STRAT4 assay could be a potential solution to overcome IHC/FISH limitations and may help to determine the best BC treatment in low-resource countries.

## Conclusion

Preliminary results revealed a high result agreement between breast STRAT4 assay and IHC/FISH or SISH, using Cape Verde FFPE tissue samples. Therefore, a point-of-care mRNA BC STRAT4 assay as an automated, quick, and easy-to-use assay, that allows surrogate BC sub-typing, may be an alternative in laboratories unable to provide quality and/or cost-efficient IHC services but that have this technology available.

## Conflicts of interest

The authors declare that there is no conflict of interest.

## Disclaimer

Where authors are identified as personnel of the International Agency for Research on Cancer/World Health Organization, the authors alone are responsible for the views expressed in this article and they do not necessarily represent the decisions, policy or views of the International Agency for Research on Cancer/World Health Organization.

## Author contributions

This study was conceptualised and designed by Pamela CC Borges, André Lopes Carvalho, and Lúcio Lara Santos. It was written by Pamela CC Borges and Lúcio Lara Santos. STRAT4 assay was performed by Pamela CC Borges and Dylan Ferreira. Acquisition of data was carried out by Pamela CC Borges, Hirondina B Spencer, and Victor Costa. Analysis and interpretation of histology features were performed by Carla Barbosa, Antónia Furtado, Maria Conceição Leal and Carlos Lopes. Analysis and interpretation of data were done by Pamela CC Borges and Lúcio Lara Santos. Pamela CC Borges and Lucio Lara Santos drafted, and Isabel dos Santos Silva and André Lopes Carvalho revised the article for important intellectual content. All authors read and agreed to the final version of this manuscript.

## Figures and Tables

**Table S1.** IHC results for hormone receptors (ER, PR, HER2, and Ki67) and BC STRAT4 assay results for mRNA expression of genes related to hormone receptors (ESR1, PGR, ERBB2, MKi67).

Sample code	Results		
	IHC		Xpert Strat4
	IMP	LAP	
1	----------------	ER: Pos (90%–100%) PR: Pos (40%–50%) HER2: Neg (0%) Ki67: Neg (<15%)	ESR1: Pos PGR: Pos ERBB2: Neg MKi67: Neg
2	ER: Pos (75%–100%) PR: Pos (75%–100%) HER2: Neg (0%) Ki67:Pos(>15%)	ER: Pos (90%–100%) PR: Pos (40%–50%) HER2: Neg (0%) Ki67: Pos (>30%)	ESR1: Pos PGR: Pos ERBB2: Neg MKi67: Pos
3	ER: Pos (75%) PR: Neg (0%) HER2: Pos (3+) Ki67: Pos (≥20%)	ER: Pos (90%–100%) PR: Neg (0%) HER2: Pos (3+) Ki67: Pos (15%–30%)	ESR1: Pos PGR: Neg ERBB2: Pos MKi67: Pos
4	ER: Pos (82%) PR: Pos (64%) HER2: Neg (0%) Ki67: Pos (21%)	ER: Pos (90%–100%) PR: Pos (80%–90%) HER2: Neg (0%) Ki67: Pos (>30%)	ESR1: Pos PGR: Pos ERBB2: Neg MKi67: Pos
5	ER: Neg (0%) PR: Neg (0%) HER2: Neg (1+) Ki67: Pos (30%–40%)	ER: LowInt (1%–10%) PR: Neg (0%) HER2: Neg (0%) Ki67: Pos (>50%)	ESR1: Neg PGR: Neg ERBB2: Neg MKi67: Pos
6	----------------	ER: Pos (40%–50%) PR: LowInt (1%–10%) HER2: Pos (3+) Ki67: Pos (>50%)	ESR1: Pos PGR: Neg ERBB2: Pos MKi67: Pos
7	ER: Neg (0%) PR: Neg (0%) HER2: Neg (0%) Ki67: Pos (>70%)	ER: Neg (1%) PR: Neg (0%) HER2: Neg (0%) Ki67: Pos (>50%)	ESR1: Neg PGR: Neg ERBB2: Neg MKi67: Pos
8	ER: Pos (50%) PR: Pos (50%) HER2: Neg (0%) Ki67: Pos (15%–20%)	ER: Pos (90%–100%) PR: Pos (80%–90%) HER2: Neg (0%) Ki67: Pos (>30%)	ESR1: Pos PGR: Pos ERBB2: Pos MKi67: Pos
9	----------------	ER: Pos PR: Neg HER2: Neg Ki67: Neg (<5%)	ESR1: Pos PGR: Pos ERBB2: Neg MKi67: INDETER
10	ER: Neg (0%) PR: Neg (0%) HER2: Pos (3+) Ki67: Pos (≥15%)	ER: Neg (0%) PR: Neg (0%) HER2: Provavel + Ki67: Pos (15%–30%)	ESR1: INVALID PGR: INVALID ERBB2: INVALID MKi67:INVALID
11	ER: Neg PR: Neg HER2: Neg (0%) Ki67: Pos (70%)	----------------	ESR1: Neg PGR: Neg ERBB2: Neg MKi67: Pos
12	ER: Neg PR: Neg HER2: Pos (3+) Ki67: Pos (10%–15%)	----------------	ESR1: Neg PGR: Neg ERBB2: Pos MKi67: Neg
13	ER: Pos (80%–90%) PR: Pos (80%–90%) HER2: Neg (1+) Ki67: Pos (≥15%)	----------------	ESR1: Pos PGR: Pos ERBB2: Neg MKi67: Pos
14	ER:Pos (25%–50%) PR: Neg HER2: (2+)/SISH: Neg. Ki67: Pos (25%–35%)	----------------	ESR1: Neg PGR: Neg ERBB2: Neg MKi67: Neg
15	ER:Pos (25%–50%) PR: Neg (10%–15%) HER2: Neg (1+) Ki67: Pos (≥15%)	----------------	ESR1: Neg PGR: Neg ERBB2: Neg MKi67: Neg
16	ER:Pos (100%) PR: Pos (20%) HER2: Neg Ki67: Pos (30%)	----------------	ESR1: Pos PGR: Pos ERBB2: Neg MKi67: Pos
17	ER:Pos (70%–80%) PR: Pos (20%–30%) HER2: Neg (1+) Ki67: Pos (≥15%)	----------------	ESR1: Pos PGR: Neg ERBB2: Neg MKi67: Pos
18	ER: Pos (50%–75%) PR: Neg HER2: Neg Ki67: Neg (1%)	----------------	ESR1: Pos PGR: Neg ERBB2: Pos MKi67: Neg
19	ER: Pos (30%–40%) PR: Pos (30%–40%) HER2: Neg (0%) Ki67: Pos (20%)	----------------	ESR1: INVALID PGR: INVALID ERBB2: INVALID MKi67: INVALID
20	ER: Pos (80%) PR: Pos (25%) HER2: Neg (0%) Ki67: Neg (2%)	----------------	ESR1: Pos PGR: Pos ERBB2:Neg MKi67: INDETER
21	ER: Pos (75%) PR: Pos (5%) HER2: Neg (0%) Ki67: Pos (≥15%)	----------------	ESR1: Pos PGR: Pos ERBB2:Neg MKi67: Pos
22	ER: Pos (80%–90%) PR: Pos (5%–10%) HER2: Neg (1+) Ki67: Neg (10%)	----------------	ESR1: Pos PGR: Pos ERBB2: Neg MKi67: INDETER
23	ER: Neg PR: Neg HER2: Neg Ki67: Pos (75%)	----------------	ESR1: Neg PGR: Neg ERBB2: Neg MKi67: Pos
24	ER: Pos (90%) PR: Neg HER2: Neg (0%) Ki67: Pos (20%)	----------------	ESR1: Pos PGR: Neg ERBB2: Neg MKi67: Pos
25	ER: Pos (90%) PR: Pos (20%) HER2: Neg (1+) Ki67: Pos (60%)	----------------	ESR1: Pos PGR: Pos ERBB2: Neg MKi67: Pos
26	ER: Pos (60%–70%) PR: Pos (70%–80%) HER2: Neg (0%) Ki67: Pos ≥15%	----------------	ESR1: Pos PGR: Pos ERBB2: Neg MKi67: Neg
27	ER: Pos (25%–50%) PR: Pos (20%) HER2: (2+)/SISH: + Ki67: Not determined	----------------	ESR1: Pos PGR: Pos ERBB2: Pos MKi67: Pos
28	ER: Neg PR: Neg HER2: Neg Ki67: Not determined	----------------	ESR1: Neg PGR: IINDETER ERBB2: Neg MKi67: Pos
29	ER: Pos (55.5%) PR: Pos (76%) HER2: Neg (0%) Ki67: Neg (≤14.8%)	----------------	ESR1: Pos PGR: Pos ERBB2: Neg MKi67: Neg

LowInt: Low-intensity signal; IMP: IMP diagnostics, molecular and anatomic pathology; LAP: anatomy-pathology laboratory

a Pre-analytic problems – fixation artifacts in both IMH

b Scarce amount of tumor material

**Table S2.** BC molecular classification based on IHC and STRAT4 assay results.

Sample Code	BC classification results		IHC/STRA4 concordance
	HC	STRAT4	
1	Luminal A	Luminal A	Concordant
2	Luminal B	Luminal B	Concordant
3	Luminal B HER2+	Luminal B like HER2+	Concordant
4	Luminal B	Luminal B	Concordant
5	Triple negative	Triple negative	Concordant
6	Luminal B HER2+	Luminal B like HER2+	Concordant
7	Triple negative	Triple negative	Concordant
8	Luminal B	Luminal B like HER2+	Discordant
9	Luminal B	Luminal A	Discordant
10	HER2 enriched	INVALID	NA
11	Triple negative	Triple negative	Concordant
12	HER2 enriched	HER2 enriched	Concordant
13	Luminal B	Luminal B	Concordant
14	Luminal B	Triple negative	Discordant
15	Luminal B	Triple negative	Discordant
16	Luminal B	Luminal B	Concordant
17	Luminal B	Luminal B	Concordant
18	Luminal B	Luminal B like HERB2+	Discordant
19	Luminal B	INVALID	NA
20	Luminal A	Luminal A	Concordant
21	Luminal B	Luminal B	Concordant
22	Luminal A	Luminal A	Concordant
23	Triple negative	Triple negative	Concordant
24	Luminal B	Luminal B	Concordant
25	Luminal B	Luminal B	Concordant
26	Luminal B	Luminal A	Discordant
27	Luminal B HER2+	Luminal B HER2+	Concordant
28	Triple negative	Triple negative	Concordant
29	Luminal A	Luminal A	Concordant

NA: Not applicable

**Table 1.** Percentage of concordance of IHC and STRAT4 assay results for EE, PR, HER2 and Ki67.

ICH/STRAT4 (S4)	Total sample	S4 assay successful	S4 assay failed/ invalid	Undetermined	ICH+ & S4+	ICH− & S4−	ICH+ & S4−	ICH− & S4+	Observed agreement (%)	Cohen’s k-statistics	95% CI	Interpretation
ER/ESR1	29	27	2	0	19	6	2	0	92.59%	0.809	0.558–1	Almost perfect agreement
PR/PGR	29	27	2	1	13	11	1	1	92.31%	0.845	0.639–1	Almost perfect agreement
HER2/ERBB2	29	27	2	0	4	21	0	2	92.59%	0.757	0.442–1	Substantial agreement
Ki67/Mki67	29	24	2	3	15	3	4	0	81.82%	0.506	0.125–0.887	Moderate agreement

K' Statistics

95% CI, *p* < 0.05

**Table 2.** Percentage of concordance between BC molecular classification made based on IHC and STRAT4 assay results.

ICH/STRAT4 (S4)	Total sample	Not applicable	Observed agreement (%)	Cohen’s k-statistics	95% CI	Interpretation
BC classification	29	2	77.78	0.701	0.493–0.909	Substantial agreement

K' Statistics

95% CI, *p* < 0.05
